# Hemagglutinin Receptor Binding of a Human Isolate of Influenza A(H10N8) Virus

**DOI:** 10.3201/eid2107.141755

**Published:** 2015-07

**Authors:** Irene Ramos, Mena Mansour, Teddy J. Wohlbold, Megan E. Ermler, Ariana Hirsh, Jonathan A. Runstadler, Ana Fernandez-Sesma, Florian Krammer

**Affiliations:** Icahn School of Medicine at Mount Sinai, New York, New York, USA (I. Ramos, M. Mansour, T.J. Wohlbold, M.E. Ermler, A. Hirsh, A. Fernandez-Sesma, F. Krammer);; Massachusetts Institute of Technology, Cambridge Massachusetts, USA (J.A. Runstadler)

**Keywords:** influenza A(H10N8) virus, influenza A(H10N7), viruses, influenza virus, influenza, emerging influenza, avian influenza, hemagglutinin receptor binding, sialic acid

## Abstract

Three cases of influenza A(H10N8) virus infection in humans have been reported; 2 of these infected persons died. Characterization of the receptor binding pattern of H10 hemagglutinin from avian and human isolates showed that both interact weakly with human-like receptors and maintain strong affinity for avian-like receptors.

Human infections with avian influenza A(H10N8) virus were reported in China during the 2013–14 winter influenza season. The first patient, a 73-year old woman, became ill in November 2013 a few days after visiting a live poultry market in Jiangxi Province (*1*). Two additional patients, a 55-year-old woman and a 75-year-old man, were admitted to hospitals in the same province in January 2014 (*2*). Severe pneumonia and subsequent acute respiratory distress syndrome developed in all 3 patients; 2 of the patients died, 5 and 6 days after admission (*2*).

Epithelial cells of the human upper respiratory tract contain mostly α2,6-linked sialic acids (SAα2,6) and low levels of α2,3-linked sialic acids (SAα2,3) (*3*). Hemagglutinin (HA) of avian influenza virus strains shows preferential binding to SAα2,3 receptors, which partially accounts for the reduced ability of avian influenza strains to establish infections in humans (*3*). Interaction with SAα2,6 receptors is one of the requirements for efficient replication in the human upper respiratory tract. In addition, reduced binding to SAα2,3 facilitates respiratory droplet-based transmission in ferrets (*4*). Therefore, emerging avian influenza viruses with increased binding to SAα2,6 and reduced binding to SAα2,3 pose a major pandemic threat, and active research and surveillance to detect animal viruses with modified receptor binding are warranted.

## The Study

We analyzed the amino acid sequence of the receptor binding site of HA from the isolate A/Jiangxi-Donghu/346-1/2013 (H10-JD346; Global Initiative on Sharing Avian Influenza Data [GISAID, http://www.gisaid.org] accession no. EPI530526) from the first patient infected by influenza A(H10N8) virus. In addition, several human and avian influenza viruses (sequences from GISAID or the National Center for Biotechnology Information website) and a recent harbor seal isolate (*5*) were compared with H10-JD346 (Table). We observed that residues involved in receptor binding for H10 subtype influenza viruses suggest avian-like receptor specificity. However, we identified 2 amino acids in avian and human H10, T135 and S186, that are common in circulating human influenza viruses and were associated with changes in receptor binding in other avian influenza A virus subtypes (*6*,*7*). In accordance with this finding, Vachieri et al. found substantial levels of binding of an avian H10 HA to SAα2,6 that retained the ability to interact with SAα2,3 (*8*).

**Table:** Alignment of residues involved receptor binding of hemagglutinin of influenza A viruses*

Origin/subtype	Isolate name	Amino acid position (H3 numbering)														
		131	135	137	138	152	186	190	193	200	222	224	225	226	227	228
Human/H3N2	A/Panama/2007/1999	A	**T**	S	A	N	**S**	D	S	G	W	R	G	V	S	S
Human/H3N2	A/Texas/50/2012	T	**T**	S	A	N	G	D	F	G	R	R	N	I	P	S
Human/H3N2	A/Brisbane/10/2007	T	**T**	S	A	N	V	N	F	G	R	R	N	I	P	S
Human/H1N1	A/California/04/2009	D	V	A	A	I	**S**	D	S	T	K	R	D	Q	E	G
Human/H1N1	A/Texas/36/1991	V	V	T	S	L	**S**	D	A	A	K	R	G	Q	E	G
Human/H1N1	A/Brisbane/59/2007	T	V	A	S	L	P	D	A	A	K	R	D	Q	E	G
Avian/H1N1	A/duck/Alberta/1976	T	V	A	A	L	P	E	S	A	E	R	G	Q	A	G
Avian/H7N1	A/rhea/North Carolina/39482/1993	R	A	S	A	K	G	E	K	T	F	S	G	R	I	D
Avian/H6N1	A/mallard/Sweden/81/2002	D	V	K	A	L	P	E	T	R	A	N	G	Q	R	G
Avian (human isolate)/H5N1	A/Vietnam/1203/2004	A	V	S	A	V	N	E	K	T	K	N	G	Q	S	G
Avian (human isolate)/H7N9	A/Anhui/1/2013	R	A	S	A	K	V	E	K	K	Q	N	G	L	S	G
Avian/H10N7	A/shorebird/Delaware Bay/10/2004	N	**T**	R	A	K	**S**	E	D	L	Q	N	G	Q	S	G
Avian/H10N7	A/mallard/Interior Alaska/10BM01929/2010	N	**T**	K	A	K	**S**	E	D	L	Q	N	G	Q	S	G
Avian (seal isolate)/H10N7	A/harbor seal/Germany/1/2014	N	**T**	K	A	K	**S**	E	D	L	Q	N	G	Q	S	G
Avian (human isolate)/H10N8	A/Jiangxi-Donghu/346–1/2013	N	**T**	R	A	K	**S**	E	D	L	Q	N	G	Q	S	G

*Residues found in human H1 or H3 and in H10 hemagglutinin but not in other avian hemagglutinin sequences are shown in bold.

Given the role of receptor binding specificity of emerging influenza viruses, we analyzed the interaction of HA of the human H10-JD346 influenza A(H10N8) virus isolate in comparison with that of an avian H10N7 subtype virus. First, we used a solid-phase binding assay (*9*,*10*) and the following biotinylated glycans conjugated with a polyacrylamide (PAA) support (provided by the Consortium of Functional Glycomics [CFG]): Neu5Acα2,6Galβ1–4GlcNAcβ-PAA (6′ SLN-PAA); Neu5Acα2–6(Galβ1–4GlcNAcβ1–3)_2_β-PAA (6’sDi-LN-PAA); Neu5Acα2,3Galβ1–4GlcNAcβ-PAA (3′ SLN-PAA); Neu5Acα2–3(Galβ1–4GlcNAcβ1–3)_2_β-PAA (3′sDi-LN-PAA); and Neu5Acα2–3(Galβ1–4GlcNAcβ-sp)_3_β-PAA (3′sTri-LN-PAA). We also analyzed recombinant hexahistidine-tagged HAs (*11*) from H10-JD346, an avian H10N7 subtype strain from North America (A/mallard/Interior Alaska/10BM01929/2010; H10-mallard), a human H3N2 subtype seasonal influenza A virus (A/Panama/2007/1999; H3-P99), and an H5N1 subtype avian influenza virus from a fatal human case (A/Vietnam/1203/2004; H5-Viet).

**Figure 1:**
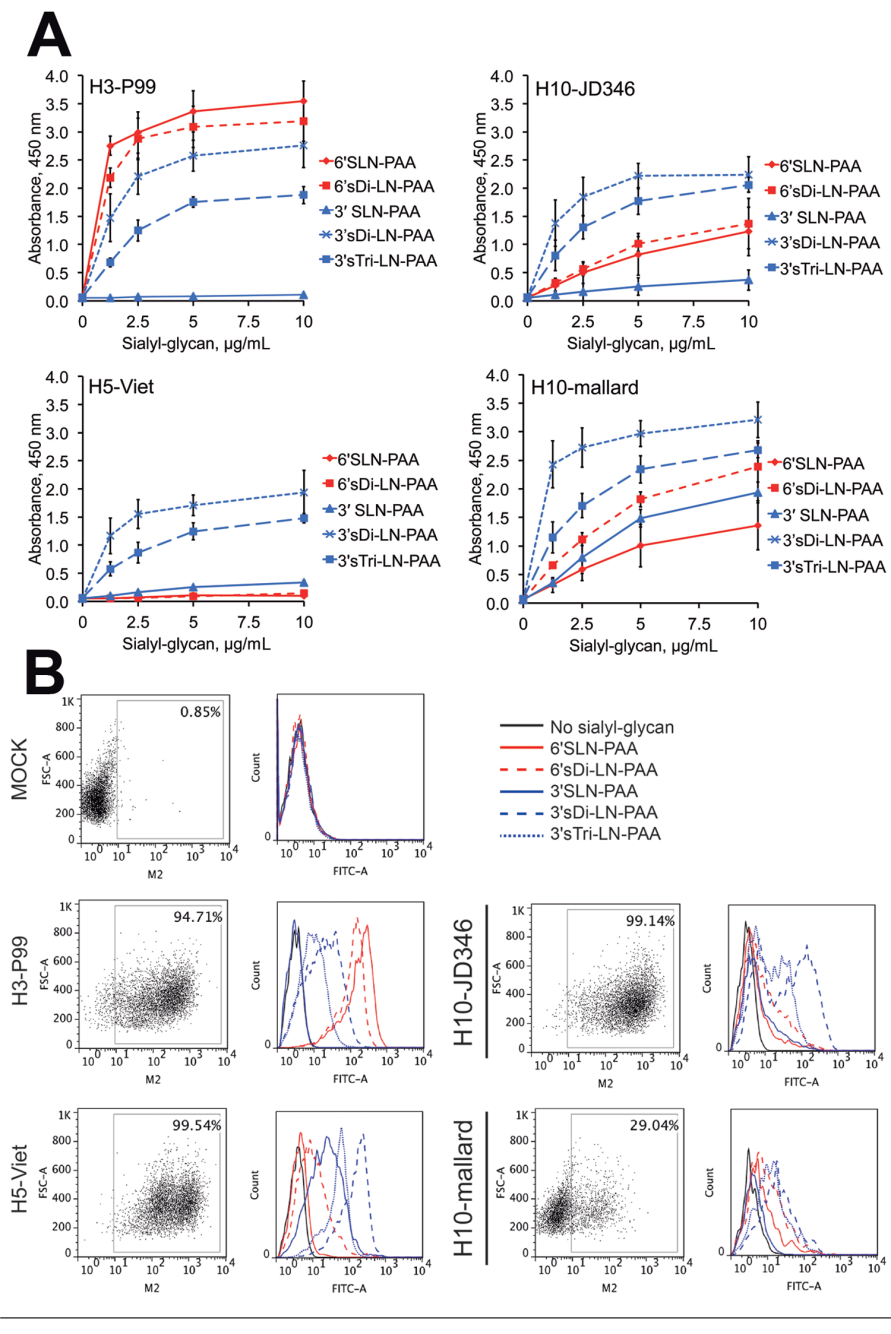
Receptor binding specificity of hemagglutinin of influenza A(H10N8) virus H10-JD346. A) Binding of recombinant hemagglutinins to glycans in a solid-phase binding assay. Results are means ± SEM of triplicate samples. PAA, polyacrylamide. B) Flow cytometry–based assay. H3-P99 (human), H5-Viet (avian origin isolated from a human case), and H10-mallard (avian) viruses were included in the analysis for comparison and as controls. Values at the top right of the dot plots indicate percentage of cells expressing matrix protein 2 (M2). FSC, forward-scattered light; FITC, fluorescein isothiocyanate.

As expected, H3-P99 bound strongly to the SAα2,6 tested, and H5 showed higher levels of binding to SAα2,3 than to SAα2,6 (Figure 1, panel A). When we analyzed H10-mallard and H10-JD346, we found a similar binding profile, which is consistent with the presence of similar amino acids affecting the receptor binding specificity (Table). Although both H10 proteins had a prevalent avian-like binding profile, low levels of binding to SAα2,6 were also observed.

To confirm this data, we used a flow cytometry–based assay and the same synthetic glycans (*9*,*10*). We infected MDCK epithelial cells with H10-JD346 virus (6:2 re-assortant with the backbone of laboratory strain A/Puerto Rico/8/1934 [PR8], which was generated as described) (*9*,*10*); H10-mallard (wild-type); human isolate H3-P99 (wild-type); and H5-Viet 6:2 (low pathogenicity reassortant with the backbone of PR8) (*9*,*10*) at a multiplicity of infection of 1. Cells were harvested 24-h postinfection and incubated with antibody against matrix protein 2 (E10), which was detected by using an antibody against IgG (Alexa 647 antibody; Invitrogen, Carlsbad, CA, USA) as a control of infection and with the sialyl-glycans (detected with streptavidin–fluorescein isothiocyanate; Jackson Laboratories, Bar Harbor, ME, USA). We determined the percentage of infected cells in each sample and gated the infected population to determine the SA binding profile (Figure 1, panel B).

H3-P99 showed high levels of binding to SAα2,6 and H5-Viet bound more efficiently to SAα2,3 than to SAα2,6, which is similar to observations with recombinant HAs in the solid-phase binding assay. H10-mallard and H10-JD346 showed similar binding profiles with preferential binding for SAα2,3 and binding to SAα2,6 slightly higher than that for the negative control.

Analysis of receptor binding of H10-JD346 and of H10-mallard with 2 independent assays indicated that the H10 subtype influenza virus interacts slightly with human-like receptors and maintains preferential binding to avian-like receptors. Consequently, these data suggest that H10 subtype influenza virus might have the ability to interact with the upper human respiratory tract, which is rich in SAα2,6 (*3*).

**Figure 2:**
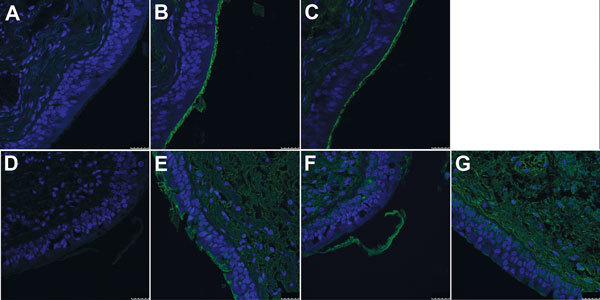
Interaction of hemagglutinin (HA) of H3-P99 (panels B and E) and H10-JD346 (panels C, F, and G) isolates of influenza A(H10N8) viruses with human trachea. Sections from 2 persons are shown (A–C and D–G). A and D, negative control staining (secondary antibody without HA). Blue indicates nuclei stained with 4',6-diamidino-2-phenylindole; green indicates HA binding. Scale bars indicate 25 μm.

**Figure 3:**
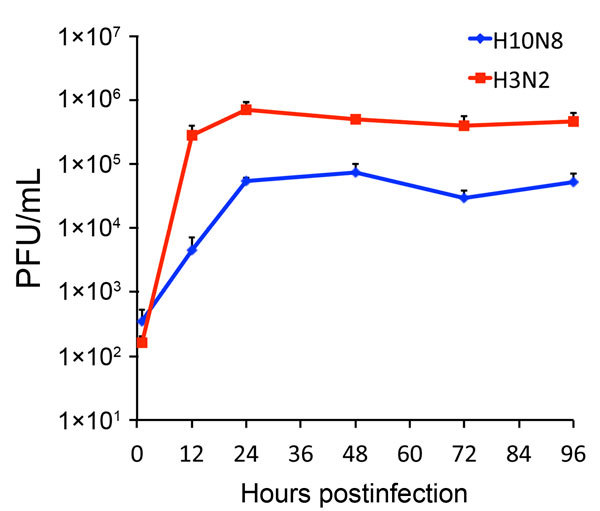
Replication of influenza A(H10N8) H10-JD346 virus in human epithelial cells. A549 cells (a human lung epithelial adenocarcinoma cell line) were infected at a multiplicity of infection of 0.1 with the H10-JD346 virus (6:2 re-assortant with the backbone of PR8) and another 6:2 re-assortant virus expressing hemagglutinin and neuraminidase genes from a human influenza A(H3N2) virus (A/Wyoming/3/2003). Cells were incubated at 37°C in Dulbecco minimal essential medium containing 0.3% bovine albumin (MP Biomedicals, Solon, OH, USA) and 1 μg/mL of tolylsulfonyl phenylalanyl chloromethyl ketone–treated trypsin (Sigma, St. Louis, MO, USA). Supernatants were collected at selected time points, and viral titers on MDCK cells were determined by using a standard plaque assay. H10-JD346 virus showed clearly lower levels of replication that that of H3N2 subtype virus.

To test this hypothesis, we precomplexed H3-P99 and H10-JD346 with primary antibody (mouse anti-His tag) and secondary fluorescent antibody, then incubated the complex with 2 human tracheal samples (*12*). As expected, H3-P99 HA bound to the surface of respiratory epithelia (Figure 2). Recombinant H10-JD346 HA also interacted with respiratory epithelia (Figure 2), which suggested that the virus might be able to attach and replicate in the human upper respiratory tract. However, the 6:2 reassortant virus H10-JD346 virus showed markedly decreased replication compared with that of an H3N2 subtype virus (PR8 6:2 reassortant) in a human lung epithelial cell line (Figure 3).

## Conclusions

HA of novel influenza A(H10N8) virus interacts with SAα2,3 and slightly with SAα2,6, at levels similar to that for an avian H10 subtype HA, and binds to cells in the human upper respiratory tract. Our findings are consistent with those of Vachiery et al. (*8*) but show some differences from those of Yang et al. (*13*) and Wang et al. (*14*), who did not detect interaction with SAα2,6 or human trachea. Variations in the experimental settings and protocols (e.g., concentration of HA or glycans used) might account for these dissimilarities.

Only 3 cases of human infections with influenza A(H10N8) viruses have been reported. However, H10N7 subtype viruses have caused conjunctivitis or mild respiratory symptoms in humans. An epidemic among seals caused by this virus subtype is currently ongoing in Europe (*5*). A study by Beare and Webster showed that ≈50% of volunteers experimentally infected with influenza A(H10N7) virus shed virus (*15*), which our data suggests might be caused by initial attachment to the upper respiratory tract. Immune responses were not detected in these volunteers, and mild, if any, symptoms developed, which indicated limited virus replication.

The low incidence of H10 influenza virus indicates a limited pandemic potential of H10N7 and H10N8 viruses. Therefore, further changes in receptor binding, as well as acquisition of genomic segments from other avian influenza virus strains through co-infection, would be required to increase fitness and transmissibility in mammals. Isolate H10-JD346 amino acid sequence had a mixture of E and K in position 627 of basic polymerase protein 2; the K627 mutation is associated with mammal adaptation (*1*). This finding highlights the need for an efficient surveillance network to track and identify possible changes, as well as extensive research to identify them and understand their functional consequences.
